# Can *Yucca schidigera* Be Used to Enhance the Growth Performance, Nutrient Digestibility, Gut Histomorphology, Cecal Microflora, Carcass Characteristic, and Meat Quality of Commercial Broilers Raised under Tropical Conditions?

**DOI:** 10.3390/ani11082276

**Published:** 2021-08-02

**Authors:** Mohamed M. Alghirani, Eric Lim Teik Chung, Danial Shah Mohd Sabri, Muhammad Nasir Jalaluddin Mohd Tahir, Nafeesa Abu Kassim, Mamat Hamidi Kamalludin, Nazri Nayan, Faez Firdaus Abdullah Jesse, Awis Qurni Sazili, Teck Chwen Loh

**Affiliations:** 1 Department of Animal Science, Faculty of Agriculture, Universiti Putra Malaysia (UPM), Serdang 43400, Selangor, Malaysia; algeranymohamed@gmail.com (M.M.A.); danialshah017@gmail.com (D.S.M.S.); nasirjalaluddin8@gmail.com (M.N.J.M.T.); mamath@upm.edu.my (M.H.K.); nazri.nayan@upm.edu.my (N.N.); awis@upm.edu.my (A.Q.S.); tcloh@upm.edu.my (T.C.L.); 2 Institute of Tropical Agriculture and Food Security, Universiti Putra Malaysia (UPM), Serdang 43400, Selangor, Malaysia; nafeesakassim@gmail.com; 3 Department of Veterinary Clinical Studies, Faculty of Veterinary Medicine, Universiti Putra Malaysia (UPM), Serdang 43400, Selangor, Malaysia; jesse@upm.edu.my

**Keywords:** antibiotic replacement, commercial broiler, production performances, steroidal saponins, *Yucca shidigera*

## Abstract

**Simple Summary:**

The broiler industry is a rapidly growing industry, particularly in developing countries, which provides a cheap source of protein via short production cycles. Despite improvements in the broiler industry, it faces several challenges such as high feed production cost, land availability, disease outbreaks, coping with rapid technology advancements, and adherence to continuous government policy change. The conditions often worsen in the tropical regions as high temperatures cause heat stress in broilers leading to negative effects on feed consumption, feed efficiency, growth rate, and mortality, resulting in a large economical loss in commercial poultry farms. As a result, any disruptions such as poor growth rate and disease outbreak could potentially harm the supply and demand, which will ultimately affect the food security of a country. Therefore, the use of antibiotics in feed has been found to increase feed efficiency and growth performance significantly in the poultry industry. Owing to the development of antimicrobial resistance in humans and livestock, the interest in using phytobiotics in veterinary medicine as an alternative to antibiotic feed additives in poultry diets is increasing.

**Abstract:**

This study aims to study the effect of *Yucca shidigera* as a phytobiotic supplementation in enhancing the production performance of commercial broilers reared under tropical environments. A total of 300 male day-old Ross 308 broiler chicks were randomly allocated into six treatment groups. Treatment 1 broilers were fed with commercial diets without antibiotics. Treatment 2 broilers were fed with commercial diets added with 100 mg/kg oxytetracycline antibiotic. Treatment 3, 4, 5, and 6 were fed with the same commercial diets added with 25, 50, 75, and 100 mg/kg *Y. shidigera*, respectively, without antibiotic. Throughout the six weeks study period, body weight and feed intake were recorded weekly for each replicate to calculate the body weight gain and feed conversion ratio. In addition, the nutrient digestibility, gut histomorphology, cecal microflora population, carcass characteristics, and meat quality were determined. The results showed significant differences (*p* < 0.05) in the growth performance, apparent ileal nutrient digestibility, gut histomorphology, carcass traits, and meat quality. Overall, T6 broilers supplemented with 100 mg/kg *Y. shidigera* demonstrated the best production performances as compared to the other treatment broilers. In summary, information from this study will be valuable for the usability of *Y. schidigera*, which could be developed as a feed additive to replace antibiotics in the poultry sector in the tropics.

## 1. Introduction

The use of antimicrobial performance enhancers has been found to increase feed conversion efficiency and growth performance significantly in the poultry industry. Nevertheless, excessive usage of antibiotics as a growth promoter in the poultry production cycle may cause drug toxicity as well as the development of antibiotic-resistant bacteria in both poultry and humans [1]. For that reason, using in-feed antibiotics in all animal feed as a promoter has been banned in developed countries owing to their harmful effects on humans [2]. Consequently, it may be necessary to explore other preventive alternatives for disease prevention and to stimulate a fast growth rate. There have been several attempts to replace antibiotics with better alternatives, namely prebiotics, probiotics, toxin binders, phytobiotics, enzymes, oligosaccharides, synbiotics, organic minerals, organic acids, and other feed additives that do not cause deleterious effects to both broilers and consumers [3].

In the last few decades, the interest in using phytobiotics in veterinary medicine as an alternative to antibiotic feed additives in diets for poultry has increased [4]. For example, *Yucca shidigera*, a native American herb, is widely used as an alternative feed additive in livestock feeds to replace antibiotics [5]. Supplementation of *Y. shidigera* in the poultry’s diet has been proven to exhibit a favorable impact on the growth rate and feed conversion of broilers [6]. This is attributed to the steroidal saponins present in the plant, which contribute to the emulsification of oil fats, promotion of their digestion, and the absorption of vitamins and minerals leading to positive effects in poultry. Furthermore, the mechanism of saponins that increase the penetrability of intestinal mucosal cells, inhibit active mucosal transport, as well as assisting the uptake of materials that are normally not absorbed could help stimulate the growth performances and immune responses [6,7,8]. Additionally, supplementation of saponin extracts was also found to decrease urea in the blood, ammonia production, and odors from poultry excreta [7].

Essentially, phytogenic feed additives such as *Y. shidigera* containing steroidal saponins could be used effectively as an alternative to antibiotics owing to their promising effects on overall production and health. Despite saponins being considered as a deleterious compound for ruminants due to the hepatic toxicity effect, steroidal saponins have been commercialized as health foods for human consumption and potential application in animal nutrition [9]. The inclusion levels of *Y. shidigera* extract or herb ranging between 125 and 225 mg/kg in poultry diets were found to be beneficial for the growth and health performances of broiler chickens [6,7]. Another study concluded that lower and higher levels of supplementation would affect broilers differently, where 100 and 200 mg/kg of *Y. shidigera* extract improved the growth performance and immune properties, respectively [8]. However, there is no precise and consistent information regarding the optimum concentration of *Y. shidigera* application for poultry in tropical climate. Therefore, the purpose of this study was to determine the effect of different concentrations of *Y. schidigera* supplementation on the growth performance, nutrients digestibility, gut histomorphology, cecal microflora, carcass characteristic, and meat quality of commercial broilers reared under hot and humid conditions.

## 2. Materials and Methods

### 2.1. Broilers and Husbandry

A total of 300 male day-old broiler chicks (Ross 308) purchased from a local hatchery were weighed and randomly allocated into six treatment groups consisting of five replications for each treatment, with 10 broilers in each replicate in a completely randomized design (CRD) experimental model. The broilers were reared on wired flooring battery cages measuring 118 cm × 88 cm × 45 cm with the stocking density of 10 birds/cage in an open-sided house for 42 days. Throughout the study period, the mean temperature and relative humidity were 29 °C and 79%, respectively, and continuous light was maintained. All chicks were vaccinated against infectious bronchitis (IB) and Newcastle disease (ND) on day 7, and with infectious bursal disease (IBD) on day 14. Feed and freshwater were available ad libitum.

### 2.2. Diets

The broilers were fed with commercial diets containing corn and soybean meal as the basal diet in crumble form. All feed was purchased from a local distributor (CP Holdings Malaysia Sdn. Bhd.). Starter diet was used from day 1 until day 21, while finisher diet was used from day 22 to day 42. Treatment 1 (negative control) broilers were fed with commercial diets without antibiotics. Treatment 2 (positive control) broilers were fed with commercial diets added with 100 mg/kg oxytetracycline antibiotic. Treatment 3, 4, 5, and 6 were fed by the same commercial diets added with 25, 50, 75, and 100 mg/kg *Y. shidigera* without antibiotic. The *Y. shidigera*-extracted powder containing not less than 60% saponins was purchased from Xi’an Longze Biotechnology Co., Ltd., Xi’an, China. The nutrient content of both starter and finisher diets supplemented with *Y. schidigera* at different concentrations are presented in Table 1. The level of dry matter (DM), crude fiber (CF), crude protein (CP), ether extract (EE), and ash content were carried out based on the method described by the AOAC International [10].

### 2.3. Growth Performance

Throughout the six weeks study period, body weight (BW) and feed intake (FI) were weekly recorded for each replicate using a digital weighing scale with a measurement accuracy of two decimal points (Mettler Toledo Industrial Scale, BBA211 series, Greifensee, Switzerland) to measure body weight gain (BWG) and feed conversion ratio (FCR) for determining the growth performance. Then, a total of ten birds (two birds from each replicate) were selected randomly and slaughtered on day 21 and 42 from each treatment group, for the determination of nutrient digestibility, gut histomorphology, cecal microflora population, carcass characteristics, and meat quality. The broilers were slaughtered according to the Halal slaughter procedure at the Department of Animal Science abattoir, Faculty of Agriculture, UPM.

### 2.4. Nutrient Digestibility

Titanium dioxide (TiO_2_) at 300 mg/kg was added to the starter and finisher diets three days before slaughtering as an indigestible marker. After slaughtering, the ileal content was collected and stored at −20 °C for further analyses of nutrient content [11]. The TiO_2_ in the digesta and the feed were determined by digesting the samples in sulphuric acid (7.4 M) and reacting with hydrogen peroxide, and then measuring the absorbance by using a 410 nm spectrophotometer [11]. Apparent ileal digestibility (AID) of dry matter (DM), crude fiber (CF), crude protein (CP), ether extract (EE), and ash were measured using titanium marker ratios in diet and ileal content according to Hashemi et al. (2014) using the following formula: AID of nutrient = 100 − [(% TiO_2_ in feed/% TiO_2_ in ileal content) × (% of nutrient in ileal content/% nutrient in feed × 100].

### 2.5. Gut Histomorphology

In order to study the gut histomorphology, 5 cm of each duodenum, jejunum, and ileum were collected, flushed with 10% neutral buffered, and preserved in formalin solution overnight. All samples were dehydrated in a tissue processing machine and embedded in paraffin wax before excising. All intestinal samples were cut at 4 mm on a slide, stained by hematoxylin and eosin, mounted, and viewed under a Nikon DS-U2/L2 light microscope. The villi height and crypt depth were examined, captured, and determined with NIS-Elements D software. The villi height was measured from tip to crypt transition, whereas the crypt depth was estimated at the invagination between two villi. This procedure was conducted according to an established method at the Histopathology Lab, Faculty of Veterinary Medicine, UPM [12].

### 2.6. Cecal Microflora Population

After slaughtering on day 21 and 42, the cecal content was also collected from each treatment group and sent immediately to the Microbiology Lab, Faculty of Veterinary Medicine, UPM for isolation and identification of micro-organisms, salmonella identification, standard plate count, and coliform count [13]. The viable colonies of the respective bacteria were counted and expressed as the log 10 of colony-forming units (cfu)g^−1^ of cecal content.

### 2.7. Carcass Characteristic

After slaughtering, all the following carcasses parameters were manually dissected and recorded: final live weight, kill-out weight, de-feathered weight, dressing percentage, the weight of the breast muscle (both left and right pectoralis major and minor), drumsticks, wings, head, shank, gastrointestinal tract, heart, liver, full, and empty gizzard [14].

### 2.8. Meat Quality Analysis

The determination of pH, color, drip loss, and cooking losses were estimated according to previous established procedures [14].

For pH determination, the right pectoralis major (breast muscle) and right soleus muscle (drumstick) were collected and snap-frozen in liquid nitrogen (−195 °C) and stored at −80 °C to maintain the meat’s pH properties before further analysis. The samples were crushed with mortar and pestle after 24 h and then homogenized. A portable pH meter (Mettler Toledo, AG 8603, Greifensee, Switzerland) was then used to determine the pH. The pH meter was calibrated with a pH 4.0 buffer and then with a pH 7.0 buffer prior to use.

For the determination of color, a color flex spectrophotometer (Hunter Lab, Reston, VA, USA) was used for this purpose, using 30 g of breast and drumstick muscle from each replicate in each treatment group. L* (lightness), b* (yellowness), and a* (redness) were provided by the color flex spectrophotometer according to the gross appearance of the color of the meat samples.

For drip loss, around 40 g of breast and drumstick samples from each treatment group were weighed and recorded as the initial weight (W1). All samples were sited in plastic bags wrapped in a vacuum and kept at 4 °C in the cooler. Then the samples were taken out from the bags after 24 and 48 h and the final weight was taken (W2). The proportion of drip loss was measured according to the following formula: (W1 − W2)/W1 × 100 to determine the drip loss.

For cooking loss, around 30 g of breast and drumstick muscle from each replicate in each treatment group were collected and weighed as the initial weight (W1). Then the samples were placed in plastic vacuum-packed bags and completely immersed for 20 min in a water bath at 80 °C. Then, samples were taken out from the water bath to allow the bags to cool down before weighing as the final weight (W2). The loss of cooking was estimated by this formula: (W1 − W2)/W1 × 100.

After the cooking loss was measured, the cooked meat was subjected to tenderness analysis. The test was based on the mechanical force (kg) require for shearing a cooked meat sample’s muscle fibers. The textural evaluation of the tenderness of cooked poultry meat was performed using a Volodkovich blade set with the TA HD plus^®^ texture analyzer (Stable Micro Device, Surrey, UK), which was balanced by weight at 5 kg, and had a return distance of 10 mm by height and a blade speed set at 10 mm/sec. Each sample was split into three stripes (1 × 1 × 2 cm^3^) that were cut as parallel as possible to the direction of the muscle fibers. Every sample was sheared via the Volodkevitch jaw (stainless steel probe shaped like an incisor) on the texture analyzer (Stable Micro System, Surrey, UK).

### 2.9. Statistical Analysis

The analysis of all data was done using one-way analysis of variance (ANOVA) based on the completely randomized design model, using Statistical Analysis System (SAS, 2012, SAS Institute, Cary, NC, USA ), and Tukey post-hoc test was used to estimate the significant difference among treatment groups. Results were considered significant at *p* < 0.05.

## 3. Results

### 3.1. Growth Performance

Table 2 presents the growth performance results of broilers on day 21 and 42. There was no significant difference (*p* > 0.05) in the growth performance parameters during the starter phase. However, significant differences (*p* < 0.05) were noted in the final body weight, body weight gain, and cumulative FCR during the finisher phase. T6 broilers supplemented with 100 mg/kg *Y. shidigera* demonstrated the highest final body weight and body weight gain as well as the lowest FCR as compared to the other treatments, specifying a better growth performance.

### 3.2. Ileal Nutrient Digestibility

The results on the AID of broilers on day 21 and 42 are shown in Table 3. There were significant differences (*p* < 0.05) in all the parameters during both starter and finisher phases. Significantly higher CP, CF, and EE were demonstrated in T6 broilers compared to T1–T5 treatments. Besides, the DM and ash were significantly lower in the T6 treatment as compared to the other treatments during the starter phase. Likewise, similar findings were exhibited during the finisher phase indicating that T6 broilers supplemented with the highest concentration of *Y. shidigera* at 100 mg/kg have a better ileal nutrient digestibility.

### 3.3. Gut Histomorphology

The small intestinal villus height and crypt depth measurements of broilers on day 21 and 42 are represented in Table 4. There were significant differences (*p* < 0.05) in the villus height and crypt depth of the duodenum, jejunum, and ileum among treatment broilers. Overall, 100 mg/kg of *Y. shidigera* supplementation was found to increase the villus height while decreasing the crypt depth in the duodenum, jejunum, and ileum of T6 broilers during both starter and finisher phases. T6 broilers had longer villus and shorter crypt depth as opposed to other treatment broilers, which could be an indicator of better gut health.

### 3.4. Cecal Micro-Organisms’ Population

Table 5 documents the cecal micro-organisms’ population of broilers on day 21 and 42. At 21 day-old, *Escherichia coli* and *Bacillus *sp. were detected in all treatment groups. However, *Enterococcus faecalis* was only found in T1 broilers, which were not supplemented with any additives. Nevertheless, T6 broilers supplemented with the highest concentration of *Y. schidigera* had the highest standard plate count and coliform count as compared to the other treatments. On the other hand, there was no variation in the cecal micro-organisms’ population at 42 day-old. *E. faecalis*, *E. coli*, and *Bacillus *sp. were isolated and identified in all treatment groups, plus the standard plate count and coliform count were almost similar. *Salmonella *sp. was also not detected in all treatment broilers during both starter and finisher phases.

### 3.5. Carcass Characteristics

The effects of different *Y. schidigera* supplementations on the carcass characteristics of broilers on day 21 and 42 are displayed in Table 6 and Table 7. There were significant differences (*p* < 0.05) in the final live weight, kill-out weight, de-feathered weight, carcass weight, dressing percentage, breast, drumstick, and wing weight among treatment groups at 21 day-old and 42 day-old. The neck weight only showed a significant difference at 42 day-old. Likewise, T6 broilers supplemented with 100 mg/kg of *Y. schidigera* revealed the highest values in contrast to other treatment broilers at 21 day-old and 42 day-old, signifying the best carcass traits. There were no significant differences (*p* > 0.05) in the other parameters. Nevertheless, heavier carcass characteristics were also observed in T6 broilers.

### 3.6. Meat Quality

Table 8 and Table 9 report the effects of different *Y. schidigera* supplementations on the breast and drumstick quality of broilers on day 21 and 42. On both terms, there were significant differences (*p* < 0.05) in the cooking loss, muscle pH, drip loss at 24 h, and drip loss at 48 h in the breast and drumstick muscle among treatment groups. Color L* (lightness) only demonstrated a significant difference in the breast muscle at 21 day-old. Generally, T6 broilers depicted the highest pH value, as well as the lowest cooking loss and drip loss values, which indicate better meat quality as opposed to the other treatments. There were no significant differences (*p* > 0.05) in the other parameters.

## 4. Discussion

Growth performance is considered as a significant parameter to measure the production of broilers which could be affected by numerous factors such as the environment and nutrition [14]. In the present study, T6 broilers supplemented with 100 mg/kg *Y. shidigera* extract showed a better growth performance throughout the 42 days study period. The result was in line with previous studies that reported that the growth performance of broilers was improved with supplementation of *Y. schidigera* at different concentrations ranging from 125 to 225 mg/kg [6,7]. The growth-promoting effects of *Y. schidigera* were attributed to the presence of steroidal saponins, which have a positive effect on the digestive tract through activation of digestive enzymes and enhancement of the villus height. These characteristics will then facilitate the absorption of nutrients and other substances that are normally not absorbed [9]. In addition, the polyphenols compound present in *Y. schidigera* also plays a major role in anti-inflammatory, antimicrobial, and antioxidant activity, as well as free-radical hunting characteristics and immune enhancement, which all improve the growth performance of broilers [8]. Saponins contained in *Y. schidigera* have been described as an anti-nutritional agents in most cases with regard to their biological function [15,16]. Nonetheless, the beneficial effects of *Y. shidigera* were greatly affected by the level of inclusion and the amount of steroidal saponins extracted. According to a previous study, 100 mg/kg of *Y. shidigera* extract containing more than 40% of steroidal saponins improved the feed efficiency, while 200 mg/kg of supplementation mainly acted on the antioxidant and immune properties [8]. As a result, lower concentrations of *Y. shidigera* supplementation as applied in the present and previous studies did not have any significant effect on the growth performance of broilers and this may be due to the inadequate amount of additives to stimulate the metabolism.

The use of phytogenic feed additives such as *Y. shidigera* containing steroidal saponins was found to improve intestinal health, reducing microbial pressure and stabilizing bowel health while preventing intestinal disorder, which could lead to improved nutrient digestibility and absorption [17]. The intestinal presence of saponins has previously been shown to influence the permeability of the membrane and the cellular transport of molecules such as macromolecules. In the current study, T6 broilers demonstrated the highest CP, CF, and EE digestibility throughout the study period. Similarly, previous work has documented significantly higher energy and protein values indicating a better energy utilization and protein digestibility in broiler chicks supplemented with *Y. schidigera* [7]. *Y. schidigera* extract supplementation can affect energy metabolism by modulating hormone secretions and depressing energy compounds in the organism [18]. Additionally, modified mRNA levels of peptide and amino acid transporters were exhibited in groups fed with phytobiotic feed supplementations which may improve the absorption of protein in the small intestine [19]. Moreover, significantly higher ether extract digestibility could be caused by the emulsifying properties (stabilizing water or oil emulsions) of saponins and their role in making monoglycerides more soluble. Therefore, dietary supplementation with saponins will result in the emulsification of oil fats and ultimately boost digestion, which was observed in the current study. Conversely, the inclusion of *Y. schidigera* in broiler diets showed significantly lower digestibility of DM and ash in the present study. The lower DM digestibility of phytogenic ingredients can be due to the higher composition of fiber in the diet [20].

Gut histomorphology can be affected differently by the bacteriostatic effect as a result of changing gut pH, epithelial cell proliferation, acid secretion, gastrin production, and nutrient alterations [21]. For instance, the use of phytogenic feed additives such as saponins was found to improve intestinal health, reducing microbial pressure, stabilizing bowel health, as well as preventing intestinal disorders [22]. In the current study, the villi height of the duodenum, jejunum, and ileum increased linearly with increasing levels of *Y. shidigera* supplementation in the broiler diets during both starter and finisher phases. Villi may be developed in response to lumen conditions reflecting the dynamic inner environment of the broiler gut or due to the antioxidant ability of saponins that may alter intestinal histomorphology [23]. The absorptive surface of the intestine is expanded when the height of intestinal villi increases, leading to an improved nutrient absorption as well as better feed efficiency of broilers as shown in the present study. In line with this, few studies also observed an increase in villus height of the duodenum, jejunum, and ileum, as well as a heavier body weight in broilers that were supplemented with *Y. shidigera* extracts [24,25]. On the other hand, broilers supplemented with 100 mg/kg of *Y. shidigera* had the shortest crypt depth along the small intestine. Shorter crypt depths occur as a result of a slower turnover in the intestinal mucosa or enterocytes differentiating activity resulting in lower maintenance requirements [12]. As the turnover is slower, lower maintenance is required for villi regeneration and, for that reason, more energy is focused on the growth performance, where heavier body weight was displayed by T6 broilers. On the contrary, T1 broilers not supplemented with any additives had the shortest villi and deepest crypt depth, which could be attributable to the continuous effect of inflammation from pathogens or toxins that may fasten the tissue turnover [21].

Numerous studies have reported that some plants have antimicrobial properties when they are complemented in the poultry diet [26]. Previous work has suggested that the *Y. schidigera* plant has antimicrobial properties because of the high content of saponins [27]. In the current study, there were differences in the type of micro-organisms, standard plate count, and coliform count in the cecal content of broilers during the starter phase. *E. faecalis* was only isolated and identified in T1 broilers not supplemented with any additives, whereas the organism was not detected in broilers supplemented with antibiotic and *Y. shidigera*. *E. faecalis* is a normal flora bacteria of poultry with nonmotile, Gram-positive, and coccoid characteristics. However, this bacteria is considered an opportunistic pathogen that can cause severe clinical signs in all ages of birds, especially in embryos and brooding chicks [28]. The results from the current study proved that *Y. shidigera* has antimicrobial effects against pathogenic bacteria such as *E. faecalis*. The results were consistent with previous work that reported 5 to 10% of *Y. schidigera* extract was sufficient to inhibit the growth of *E. avium* and *E. faecalis* in poultry manure [27]. This could be due to the presence of steroidal saponins that are poorly absorbed, hence altering the microbial fermentation in the gastrointestinal tract. On top of that, plant extracts such as *Y. shidigera* could provide beneficial micro-organisms an advantage in nutrient competition and eventually improve the nutrient digestibility of broiler chickens. Supporting this idea, the addition of plant preparations could help decrease the level of intestinal pH providing an optimum environment for lactic acid bacteria proliferation in the ileum and cecal contents while significantly decreasing the *Clostridium perfringens* and *E. coli* counts [13]. For that reason, the highest standard plate and coliform count observed in T6 broilers could be attributed to the proliferation of beneficial microflora in the gut. In contrast, there were no significant differences in the microbial results during the finisher phase in the current study. This is because the coliform count, lactose-negative enterobacteria, and lactic acid bacteria in the cecal content would reduce gradually as the broilers grew older [29].

There is a strong relationship between body weight and carcass weight, as well as between carcass weight and dressing percentage [30]. Phytobiotics could be practiced to enhance the carcass traits while adding value to poultry products due to their antimicrobial and antioxidant characteristics [23]. Moreover, supplementing plant extracts to the poultry diet was reported to improve the breast muscle percentage of the eviscerated carcass by 1.2% [31]. In the present study, a numerical improvement in the live weight was observed in broilers fed with *Y. schidigera* supplementation, which positively affects the carcass characteristics. Supplementing 100 mg/kg of *Y. schidigera* has proven to improve the carcass yield, particularly the carcass weight, dressing percentage, and breast, drumstick, wing, and neck weights. Previous research demonstrated similar results where the *Y. shidigera* treatment broiler group exhibited higher evisceration weight, breast meat yield, and thigh meat yield [7]. The beneficial effect of the steroid saponins present in *Y. schidigera* on gut histomorphology and nutrient absorption can be linked with the positive findings in the present study [32]. A similar trend reported greater breast and thigh meat yield in broilers as well as other species of livestock fed with diets containing saponins [33]. Nonetheless, some studies did not find any substantial change in the carcass characteristics of broilers fed with either a saponin-containing diet or *Y. shidigera* supplementation [7,34]. The differences between the previous studies and the present work may be attributable to the differences between the strains of bird used, the environment, type of feed, source of saponins, and the amount of supplementation applied in those studies.

Phytobiotics could be applied during heat stress conditions to maintain or increase the meat quality of broilers, thanks to their antioxidant activity [35]. Furthermore, feeding plant secondary compounds (for example, saponins) can positively affect several quality parameters of meat in livestock [36]. In the current study, T6 broilers supplemented with the highest *Y. schidigera* demonstrated a superior meat quality during the starter and finisher phases by influencing the muscle pH, cooking loss, and drip loss of both breast and drumstick muscle. Referring to one study, prolonged time secretion of corticosterone under chronic heat stress may have detrimental effects on the breakdown of muscle due to gluconeogenesis [30]. The accelerated lactic acid production induces rapid pH decline while the body temperature is high, which results in a PSE-like conditions in meat. Nevertheless, steroidal saponins in *Y. schidigera* demonstrating antioxidant activity to prevent, delay, and protect against cell deterioration could be applied against heat stress effect to maintain the pH value which was observed in T6 broilers. Thus, a higher pH of poultry meat will then lead to a decrease in cooking loss and drip loss [30]. Supplementing 100 mg/kg *Y. schidigera* in the current study was also found to assist in reducing the cooking loss and drip loss significantly by improving water holding capacity (WHC), and this again could be attributed to the antioxidant property of saponins [35]. Oxidation creates free radicals that facilitate the oxidation of myofibril and consequently decrease muscle WHC. This was confirmed by another study that found that triterpenoid saponins fed to either broiler or lamb could help retain the lipid oxidative stability in the meat [37]. Other saponins sources (for example, Quillaja saponins) have also been shown to exhibit strong antioxidant activity [38].

## 5. Conclusions

Therefore, it can be concluded that T6 broilers supplemented with *Y. shidigera* extract showed an improved growth performance, ileal nutrient digestibility, gut health, carcass characteristics, and meat quality as opposed to the other treatment broilers. In addition, the cecal micro-organisms’ population was also increased during the starter phase. Based on the results obtained, 100 mg/kg of *Y. shidigera* supplementation can be recommended as an alternative to antibiotic growth promotant in stimulating the production performances of commercial broilers reared under tropical conditions.

## Figures and Tables

**Table 1:** Nutrient content of broiler diets supplemented with *Y. schidigera* at different concentrations.

Parameters	Treatments					
	T1	T2	T3	T4	T5	T6
Starter diet (1–21 day)						
Metabolizable energy (MJ/kg)	13.41 ± 0.07	13.67 ± 0.06	13.60 ± 0.06	13.76 ± 0.01	13.61 ± 0.13	13.62 ± 0.19
Dry matter (%)	89.77 ± 0.23	90.77 ± 0.53	90.33 ± 0.52	91.33 ± 0.20	90.57 ± 0.72	90.43 ± 0.87
Crude protein (%)	25.10 ± 0.46	25.57 ± 0.15	25.17 ± 0.52	24.90 ± 0.30	25.07 ± 0.27	26.13 ± 0.32
Crude fiber (%)	3.40 ± 0.15	3.40 ± 0.06	3.47 ± 0.09	3.13 ± 0.09	3.23 ± 0.37	2.97 ± 0.27
Ether extract (%)	6.85 ± 0.49	7.20 ± 0.29	7.35 ± 0.20	7.15 ± 0.09	7.20 ± 0.29	7.00 ± 0.00
Ash (%)	5.90 ± 0.10	5.67 ± 0.20	5.77 ± 0.23	5.80 ± 0.10	6.00 ± 0.17	5.80 ± 0.10
Finisher diet (22–42 day)						
Metabolizable energy (MJ/kg)	13.01 ± 0.03	12.88 ± 0.10	12.90 ± 0.04	13.10 ± 0.08	12.90 ± 0.28	13.18 ± 0.04
Dry matter (%)	90.30 ± 0.00	90.20 ± 0.59	89.57 ± 0.43	90.43 ± 0.59	89.97 ± 0.67	90.43 ± 0.30
Crude protein (%)	16.33 ± 0.26	15.77 ± 0.32	16.37 ± 0.19	16.87 ± 0.29	17.00 ± 0.38	16.30 ± 0.32
Crude fiber (%)	3.95 ± 0.20	4.53 ± 0.19	3.70 ± 0.12	3.40 ± 0.38	4.50 ± 1.55	3.40 ± 0.17
Ether extract (%)	4.67 ± 0.20	4.70 ± 0.00	4.57 ± 0.13	4.70 ± 0.00	4.67 ± 0.33	5.00 ± 0.17
Ash (%)	5.43 ± 0.13	5.67 ± 0.20	5.57 ± 0.13	5.57 ± 0.13	5.43 ± 0.13	5.80 ± 0.10

Note: T1: Negative control; T2: Positive control; T3: 25 mg/kg; T4: 50 mg/kg; T5: 75 mg/kg; T6: 100 mg/kg.

**Table 2:** Effect of *Y. schidigera* supplementation on the growth performance of broilers on day 21 and 42.

Parameters	Treatments						*p* Value
	T1	T2	T3	T4	T5	T6	
21 day-old (Starter phase)							
Initial body weight (kg)	0.05 ± 0.00	0.04 ± 0.00	0.04 ± 0.00	0.05 ± 0.00	0.04 ± 0.00	0.05 ± 0.00	0.54
Final body weight (kg)	0.90 ± 0.01	0.90 ± 0.01	0.93 ± 0.01	0.91 ± 0.01	0.92 ± 0.02	0.92 ± 0.02	0.31
Body weight gain (kg)	0.85 ± 0.01	0.86 ± 0.01	0.88 ± 0.01	0.83 ± 0.02	0.87 ± 0.02	0.87 ± 0.02	0.31
Feed intake (kg)	1.17 ± 0.02	1.19 ± 0.03	1.21 ± 0.02	1.19 ± 0.04	1.16 ± 0.03	1.19 ± 0.02	0.81
Cumulative FCR	1.38 ± 0.03	1.39 ± 0.03	1.38 ± 0.03	1.43 ± 0.07	1.34 ± 0.04	1.37 ± 0.03	0.73
42 day-old (Finisher phase)							
Final body weight (kg)	1.97 ± 0.04 ^b^	2.00 ± 0.04 ^b^	2.04 ± 0.03 ^ab^	2.04 ± 0.02 ^ab^	2.05 ± 0.06 ^ab^	2.27 ± 0.11 ^a^	0.02
Body weight gain (kg)	1.92 ± 0.04 ^b^	1.95 ± 0.04 ^b^	2.00 ± 0.03 ^ab^	2.00 ± 0.02 ^ab^	2.00 ± 0.06 ^ab^	2.22 ± 0.11 ^a^	0.01
Feed intake (kg)	4.08 ± 0.11	4.30 ± 0.07	4.26 ± 0.06	4.19 ± 0.11	4.26 ± 0.10	4.22 ± 0.05	0.59
Cumulative FCR	2.12 ± 0.03 ^ab^	2.20 ± 0.02 ^a^	2.14 ± 0.04 ^ab^	2.10 ± 0.05 ^ab^	2.13 ± 0.03 ^ab^	1.92 ± 0.10 ^b^	0.03

Note: All values were expressed as mean ± SE; ^a,b^ values with superscript within row are significantly different at *p* < 0.05. FCR: Feed conversion ratio. T1: Negative control; T2: Positive control; T3: 25 mg/kg; T4: 50 mg/kg; T5: 75 mg/kg; T6: 100 mg/kg.

**Table 3:** Effect of *Y. schidigera* supplementation on the apparent ileal nutrient digestibility of broilers on day 21 and 42.

Parameters	Treatments						*p* Value
	T1	T2	T3	T4	T5	T6	
21 day-old (Starter phase)							
Dry matter (%)	67.56 ± 0.51 ^cd^	67.31 ± 0.46 ^cd^	73.16 ± 0.49 ^a^	71.46 ± 0.82 ^ab^	68.54 ± 0.69 ^bc^	64.49 ± 0.25 ^d^	0.01
Crude protein (%)	51.42 ± 0.90 ^b^	51.92 ± 0.83 ^b^	51.73 ± 0.56 ^b^	54.65 ± 0.90 ^b^	55.68 ± 0.99 ^b^	67.64 ± 0.86 ^a^	0.01
Crude fibre (%)	31.36 ± 1.21 ^a^	33.37 ± 0.35 ^ab^	34.81 ± 0.08 ^ab^	33.82 ± 1.08 ^ab^	33.09 ± 0.74 ^ab^	36.53 ± 0.32 ^a^	0.03
Ether extract (%)	73.51 ± 0.16 ^b^	75.01 ± 0.76 ^ab^	74.67 ± 0.00 ^ab^	72.89 ± 0.78 ^b^	74.04 ± 0.76 ^ab^	77.27 ± 0.65 ^a^	0.02
Ash (%)	45.19 ± 0.47 ^a^	44.38 ± 0.64 ^ab^	43.42 ± 0.22 ^ab^	45.30 ± 0.54 ^a^	45.69 ± 1.06 ^a^	41.39 ± 0.35 ^b^	0.02
42 day-old (Finisher phase)							
Dry matter (%)	65.79 ± 0.02 ^bc^	64.92 ± 0.07 ^c^	66.83 ± 0.14 ^ab^	65.22 ± 0.17 ^c^	67.22 ± 0.42 ^a^	63.09 ± 0.24 ^d^	0.01
Crude protein (%)	51.32 ± 0.55 ^d^	53.17 ± 0.33 ^cd^	53.94 ± 0.10 ^bc^	53.89 ± 0.57 ^bc^	55.69 ± 0.12 ^ab^	57.51 ± 0.02 ^a^	0.01
Crude fibre (%)	27.96 ± 0.50 ^b^	28.92 ± 1.67 ^ab^	29.67 ± 0.18 ^ab^	29.73 ± 0.34 ^ab^	29.74 ± 0.53 ^ab^	33.01 ± 0.31 ^a^	0.04
Ether extract (%)	72.67 ± 0.60 ^cd^	74.61 ± 0.31 ^bc^	75.26 ± 0.42 ^abc^	71.77 ± 0.36 ^d^	75.99 ± 0.03 ^ab^	77.85 ± 0.79 ^a^	0.01
Ash (%)	35.67 ± 0.21 ^ab^	36.64 ± 0.15 ^a^	37.84 ± 0.07 ^a^	36.95 ± 0.95 ^a^	35.56 ± 0.43 ^ab^	33.45 ± 0.28 ^b^	0.01

Note: All values were expressed as mean ± SE; ^a,b,c,d^ values with superscript within row are significantly different at *p* < 0.05. T1: Negative control; T2: Positive control; T3: 25 mg/kg; T4: 50 mg/kg; T5: 75 mg/kg; T6: 100 mg/kg.

**Table 4:** Effect of *Y. schidigera* supplementation on the small intestinal villus height and crypt depth of broilers on day 21 and 42.

Parameters	Treatments						*p* Value
	T1	T2	T3	T4	T5	T6	
21 day-old (Starter phase)							
Villi height (μm)							
Duodenum	867.00 ± 7.96 ^b^	885.68 ± 7.65 ^b^	916.18 ± 6.99 ^a^	917.30 ± 6.71 ^a^	928.15 ± 6.45 ^a^	942.40 ± 6.37 ^a^	0.01
Jejunum	463.81 ± 3.80 ^c^	472.29 ± 4.81 ^c^	501.98 ± 4.25 ^b^	512.56 ± 4.13 ^b^	530.84 ± 4.73 ^a^	547.71 ± 4.44 ^a^	0.01
Ileum	370.53 ± 4.81 ^cd^	355.35 ± 5.82 ^d^	381.40 ± 4.94 ^bc^	391.96 ± 5.15 ^ab^	402.73 ± 5.28 ^a^	412.68 ± 5.25 ^a^	0.01
Crypt depth (μm)							
Duodenum	105.16 ± 1.43 ^a^	102.38 ± 1.30 ^ab^	99.54 ± 1.34 ^abc^	99.23 ± 1.38 ^bc^	98.06 ± 1.61 ^bc^	95.93 ± 1.38 ^c^	0.01
Jejunum	100.44 ± 4.49 ^a^	88.42 ± 3.05 ^ab^	85.90 ± 3.35 ^b^	79.05 ± 2.74 ^bc^	69.17 ± 2.73 ^cd^	65.25 ± 2.35 ^d^	0.01
Ileum	88.27 ± 3.27 ^a^	74.09 ± 3.52 ^b^	71.35 ± 2.95 ^bc^	70.73 ± 3.53 ^bc^	60.41 ± 2.06 ^cd^	56.21 ± 2.35 ^d^	0.01
42 day-old (Finisher phase)							
Villi height (μm)							
Duodenum	1144.11 ± 8.03 ^c^	1153.31 ± 8.45 ^c^	1183.95 ± 6.12 ^b^	1202.30 ± 6.36 ^ab^	1209.97 ± 5.90 ^ab^	1227.40 ± 8.20 ^a^	0.01
Jejunum	655.41 ± 5.48 ^d^	660.69 ± 4.15 ^d^	688.26 ± 6.10 ^c^	705.44 ± 6.02 ^bc^	725.79 ± 4.47 ^b^	751.92 ± 4.19 ^a^	0.01
Ileum	536.47 ± 4.62 ^d^	538.05 ± 5.56 ^d^	570.14 ± 5.57 ^c^	582.55 ± 5.15 ^c^	616.70 ± 3.76 ^b^	646.72 ± 4.58 ^a^	0.01
Crypt depth (μm)							
Duodenum	145.26 ± 4.39 ^a^	144.72 ± 3.81 ^a^	137.77 ± 4.69 ^ab^	137.19 ± 3.80 ^ab^	131.26 ± 5.06 ^ab^	124.92 ± 3.39 ^b^	0.01
Jejunum	124.56 ± 3.28 ^a^	119.47 ± 4.00 ^a^	99.93 ± 3.01 ^b^	88.94 ± 2.52 ^cb^	89.30 ± 3.63 ^cb^	85.30 ± 3.94 ^c^	0.01
Ileum	97.54 ± 2.47 ^a^	93.89 ± 2.26 ^ab^	84.38 ± 1.71 ^b^	73.07 ± 2.49 ^c^	69.68 ± 2.51 ^c^	67.28 ± 2.89 ^c^	0.01

Note: All values were expressed as mean ± SE; ^a,b,c,d^ values with superscript within row are significantly different at *p* < 0.05. T1: Negative control; T2: Positive control; T3: 25 mg/kg; T4: 50 mg/kg; T5: 75 mg/kg; T6: 100 mg/kg.

**Table 5:** Effect of *Y. schidigera* supplementation on the cecal micro-organisms of broilers on day 21 and 42.

Parameters	Treatments					
	T1	T2	T3	T4	T5	T6
21 day-old (Starter phase)						
Isolation & Identification	*E. coli* (3+) *Enterococcus faecalis* (2+)	*E. coli* (3+)	*E. coli* (3+) Non-lactose fermenting *E. coli* (2+)	*E. coli* (3+) *Bacillus* sp. (2+)	Non-lactose fermenting *E. coli* (3+)	*E. coli* (3+) Non-lactose fermenting *E. coli* (2+)
Salmonella identification	Negative	Negative	Negative	Negative	Negative	Negative
Standard plate count (cfu/mL)	1.1 × 10^8^	8.5 × 10^7^	1.3 × 10^8^	1.8 × 10^8^	1.6 × 10^9^	1.4 × 10^11^
Coliform count (cfu/mL)	9.4 × 10^7^	8.7 × 10^7^	8.7 × 10^7^	1.3 × 10^8^	2.1 × 10^8^	2.3 × 10^9^
42 day-old (Finisher phase)						
Isolation & Identification	*E. coli* (2+) *Enterococcus faecalis* (1+) *Bacillus* sp. (2+)	*E. coli* (2+) *Enterococcus faecalis* (2+) *Bacillus* sp. (1+)	*E. coli* (2+) Non-lactose fermenting *E. coli* (1+) *Enterococcus faecalis* (2+) *Bacillus* sp. (2+)	*E. coli* (2+) Non-lactose fermenting *E. coli* (1+) *Enterococcus faecalis* (2+) *Bacillus* sp. (2+)	*E. coli* (2+) *Enterococcus faecalis* (2+) *Bacillus* sp. (2+)	*E. coli* (2+) *Enterococcus faecalis* (2+) *Bacillus* sp. (2+)
Salmonella identification	Negative	Negative	Negative	Negative	Negative	Negative
Standard plate count (cfu/mL)	1.5 × 10^4^	7.8 × 10^4^	1.5 × 10^4^	1.0 × 10^4^	4.6 × 10^4^	6.2 × 10^4^
Coliform count (cfu/mL)	5.1 × 10^3^	3.2 × 10^3^	5.8 × 10^3^	2.7 × 10^3^	3.6 × 10^3^	4.1 × 10^3^

Note: cfu: Colony-forming unit. T1: Negative control; T2: Positive control; T3: 25 mg/kg; T4: 50 mg/kg; T5: 75 mg/kg; T6: 100 mg/kg.

**Table 6:** Effect of *Y. schidigera* supplementation on the carcass characteristics of broilers on day 21.

Parameters	Treatments						*p* Value
	T1	T2	T3	T4	T5	T6	
21 day-old (Starter phase)							
Final live weight (g)	935.80 ± 11.19 ^b^	941.60 ± 12.48 ^b^	943.80 ± 13.59 ^b^	951.80 ± 7.84 ^ab^	962.80 ± 4.95 ^ab^	993.40 ± 9.90 ^a^	0.01
Kill-out weight (g)	891.60 ± 2.68 ^b^	907.60 ± 9.35 ^b^	914.01 ± 12.08 ^ab^	920.20 ± 4.35 ^ab^	929.60 ± 7.76 ^ab^	954.40 ± 15.33 ^a^	0.01
De-feathered weight (g)	835.60 ± 1.86 ^b^	866.60 ± 14.44 ^ab^	871.80 ± 6.49 ^ab^	878.20 ± 18.58 ^ab^	884.40 ± 14.56 ^ab^	908.00 ± 18.71 ^a^	0.03
Carcass weight (g)	655.40 ± 7.05 ^b^	659.60 ± 112.34 ^b^	663.60 ± 12.32 ^b^	672.60 ± 8.17 ^ab^	685.10 ± 5.86 ^ab^	710.60 ± 5.75 ^a^	0.02
Dressing percentage (%)	70.04 ± 0.22 ^b^	70.03 ± 0.50 ^b^	70.30 ± 0.43 ^ab^	70.66 ± 0.28 ^ab^	71.14 ± 0.30 ^ab^	71.54 ± 0.15 ^a^	0.02
Breast (g)	242.80 ± 1.36 ^c^	242.60 ± 1.50 ^c^	242.80 ± 2.56 ^c^	248.10 ± 1.48 ^bc^	251.40 ± 1.03 ^ab^	258.20 ± 2.27 ^a^	0.02
Drumstick (g)	75.80 ± 0.49 ^b^	78.01 ± 2.68 ^ab^	82.20 ± 1.39 ^ab^	82.40 ± 0.73 ^ab^	81.40 ± 1.08 ^ab^	83.20 ± 2.33 ^a^	0.03
Wings (g)	74.10 ± 2.07 ^b^	73.80 ± 3.28 ^b^	81.80 ± 1.96 ^ab^	82.40 ± 2.93 ^ab^	85.40 ± 1.21 ^a^	90.00 ± 2.86 ^a^	0.01
Head (g)	30.20 ± 0.57	30.40 ± 1.29	30.60 ± 0.82	30.80 ± 0.37	32.00 ± 0.45	33.00 ± 0.44	0.08
Neck (g)	21.80 ± 0.74	22.20 ± 0.74	20.40 ± 0.68	21.20 ± 0.37	21.40 ± 1.08	20.60 ± 0.81	0.55
Shanks (g)	36.10 ± 0.53	36.20 ± 1.83	39.80 ± 0.37	38.40 ± 2.29	38.60 ± 1.08	41.40 ± 1.36	0.10
Full gizzard (g)	27.40 ± 0.25	26.80 ± 0.51	27.60 ± 1.47	30.10 ± 1.28	28.20 ± 0.49	29.80 ± 0.58	0.09
Empty gizzard (g)	15.30 ± 0.28	15.20 ± 0.34	15.40 ± 0.26	15.60 ± 0.41	15.60 ± 0.19	15.88 ± 0.25	0.64
GIT (g)	110.80 ± 0.74	104.20 ± 5.96	106.00 ± 0.04	114.00 ± 5.34	104.80 ± 5.34	113.20 ± 1.46	0.34
Heart (g)	5.01 ± 0.01	5.30 ± 0.25	5.00 ± 0.01	5.00 ± 0.01	5.00 ± 0.01	5.40 ± 0.03	0.10
Liver (g)	23.20 ± 0.38	26.60 ± 1.21	25.40 ± 0.75	26.40 ± 1.12	25.20 ± 0.58	27.60 ± 1.66	0.10

Note: All values were expressed as mean ± SE; ^a,b,c^ values with superscript within row are significantly different at *p* < 0.05. T1: Negative control; T2: Positive control; T3: 25 mg/kg; T4: 50 mg/kg; T5: 75 mg/kg; T6: 100 mg/kg. GIT: Gastrointestinal tract.

**Table 7:** Effect of *Y. schidigera* supplementation on the carcass characteristics of broilers on day 42.

Parameters	Treatments						*p* Value
	T1	T2	T3	T4	T5	T6	
42 day-old (Finisher phase)							
Final live weight (g)	2291.80 ± 11.41 ^bc^	2264.20 ± 5.05 ^c^	2304.40 ± 12.01 ^bc^	2332.40 ± 10.51 ^b^	2415.80 ± 11.42 ^a^	2426.80 ± 16.56 ^a^	0.01
Kill-out weight (g)	2184.30 ± 10.09 ^c^	2185.80 ± 5.63 ^c^	2200.20 ± 4.51 ^c^	2213.20 ± 20.74 ^bc^	2267.40 ± 18.92 ^b^	2344.80 ± 17.91 ^a^	0.01
De-feathered weight (g)	2143.80 ± 2.99 ^c^	2138.60 ± 6.53 ^c^	2161.00 ± 4.15 ^bc^	2160.00 ± 6.41 ^bc^	2184.00 ± 10.96 ^b^	2226.20 ± 1.78 ^a^	0.01
Carcass weight (g)	1636.60 ± 6.53 ^c^	1626.80 ± 3.32 ^c^	1656.20 ± 6.75 ^bc^	1679.80 ± 3..67 ^bc^	1740.00 ± 15.77 ^b^	1752.00 ± 5.24 ^a^	0.01
Dressing percentage (%)	71.41 ± 0.07	71.85 ± 0.07	71.87 ± 0.09	72.02 ± 0.18	72.02 ± 0.33	72.20 ± 0.34	0.20
Breast (g)	633.00 ± 1.05 ^b^	630.20 ± 2.84 ^b^	631.20 ± 1.74 ^b^	634.00 ± 3.29 ^b^	638.60 ± 3.71 ^ab^	646.00 ± 0.45 ^a^	0.02
Drumstick (g)	226.80 ± 40.58 ^b^	225.20 ± 1.32 ^b^	235.80 ± 3.15 ^ab^	226.40 ± 5.74 ^b^	234.20 ± 1.81 ^ab^	245.20 ± 4.22 ^a^	0.02
Wings (g)	198.80 ± 4.75 ^b^	201.20 ± 4.21 ^b^	201.20 ± 0.49 ^b^	204.40 ± 5.23 ^b^	210.20 ± 2.15 ^ab^	222.40 ± 4.82 ^a^	0.01
Head (g)	60.60 ± 0.25	63.80 ± 2.18	61.80 ± 2.06	62.40 ± 0.25	64.20 ± 0.92	64.80 ± 1.56	0.32
Neck (g)	42.10 ± 0.45 ^b^	42.40 ± 0.68 ^b^	41.60 ± 2.27 ^b^	44.80 ± 2.25 ^b^	45.80 ± 0.86 ^b^	53.80 ± 2.40 ^a^	0.01
Shanks (g)	98.00 ± 1.27	96.60 ± 2.46	99.20 ± 1.66	101.60 ± 2.14	100.00 ± 3.46	104.00 ± 2.19	0.29
Full gizzard (g)	43.20 ± 3.25	48.80 ± 2.94	47.80 ± 5.54	41.00 ± 4.52	44.20 ± 1.36	48.40 ± 4.43	0.65
Empty gizzard (g)	31.60 ± 1.25	30.80 ± 2.46	33.80 ± 1.83	34.00 ± 1.34	30.40 ± 0.75	33.20 ± 1.11	0.43
GIT (g)	220.20 ± 3.76	200.20 ± 7.92	213.00 ± 6.41	208.20 ± 6.86	207.60 ± 6.11	217.40 ± 5.22	0.26
Heart (g)	11.00 ± 0.01	11.40 ± 0.25	11.40 ± 0.25	11.40 ± 0.25	11.40 ± 0.25	11.60 ± 0.25	0.58
Liver (g)	45.40 ± 3.44	52.20 ± 2.87	50.40 ± 1.63	55.00 ± 2.74	46.40 ± 2.64	52.00 ± 2.59	0.25

Note: All values were expressed as mean ± SE; ^a,b,c^ values with superscript within row are significantly different at *p* < 0.05. T1: Negative control; T2: Positive control; T3: 25 mg/kg; T4: 50 mg/kg; T5: 75 mg/kg; T6: 100 mg/kg. GIT: Gastrointestinal tract.

**Table 8:** Effect of *Y. schidigera* supplementation on the meat quality of broilers on day 21.

Parameters	Treatments						*p* Value
	T1	T2	T3	T4	T5	T6	
21 day-old (Starter phase)							
Breast							
Cooking loss (%)	18.08 ± 0.74 ^a^	18.29 ± 0.28 ^a^	18.38 ± 0.42 ^a^	18.14 ± 0.58 ^a^	16.44 ± 0.62 ^ab^	15.41 ± 0.36 ^b^	0.02
Muscle pH	5.88 ± 0.02 ^b^	5.86 ± 0.01 ^b^	5.93 ± 0.04 ^ab^	5.91 ± 0.02 ^b^	6.01 ± 0.05 ^ab^	6.06 ± 0.04 ^a^	0.01
Drip loss at 24 h (%)	4.31 ± 0.10 ^a^	3.85 ± 0.15 ^ab^	3.66 ± 0.27 ^ab^	3.63 ± 0.01 ^ab^	3.70 ± 0.08 ^ab^	3.23 ± 0.29 ^b^	0.02
Drip loss at 48 h (%)	6.41 ± 0.05 ^ab^	6.30 ± 0.17 ^abc^	6.91 ± 0.46 ^a^	6.31 ± 0.20 ^abc^	5.64 ± 0.09 ^bc^	5.36 ± 0.08 ^c^	0.01
Color L* (lightness)	47.30 ± 0.73 ^ab^	47.15 ± 0.40 ^ab^	46.92 ± 0.21 ^b^	47.61 ± 0.21 ^ab^	48.92 ± 0.51 ^a^	48.49 ± 0.14 ^ab^	0.02
Color a* (redness)	6.74 ± 0.43	7.03 ± 0.49	6.24 ± 0.01	6.86 ± 0.19	6.38 ± 0.08	5.94 ± 0.24	0.12
Color b* (yellowness)	20.59 ± 0.03	18.01 ± 0.42	20.21 ± 0.42	18.38 ± 0.76	18.55 ± 0.66	19.73 ± 1.16	0.06
Tenderness	422.64 ± 20.53	411.98 ± 8.59	423.88 ± 18.87	424.98 ± 8.64	424.99 ± 9.31	446.13 ± 20.31	0.76
Drumstick							
Cooking loss (%)	20.74 ± 0.47 ^ab^	18.47 ± 0.85 ^ab^	21.09 ± 0.91 ^a^	20.23 ± 0.51 ^ab^	19.42 ± 0.31 ^ab^	17.71 ± 0.71 ^b^	0.02
Muscle pH	6.34 ± 0.0 ^b^	6.37 ± 0.02 ^b^	6.33 ± 0.04 ^b^	6.35 ± 0.05 ^b^	6.41 ± 0.02 ^ab^	6.53 ± 0.03 ^a^	0.02
Drip loss at 24 h (%)	2.14 ± 0.04	2.09 ± 0.06	2.16 ± 0.08	2.07 ± 0.07	2.05 ± 0.07	1.93 ± 0.03	0.15
Drip loss at 48 h (%)	3.17 ± 0.04 ^a^	3.03 ± 0.03 ^abc^	3.17 ± 0.05 ^a^	3.12 ± 0.04 ^ab^	3.01 ± 0.02 ^bc^	2.88 ± 0.04 ^c^	0.01
Color L* (lightness)	50.18 ± 1.34	52.14 ± 0.07	50.81 ± 0.71	50.57 ± 0.65	49.68 ± 1.69	50.19 ± 1.26	0.69
Color a* (redness)	7.07 ± 0.31	7.47 ± 0.29	8.28 ± 0.57	8.16 ± 0.54	6.49 ± 0.24	8.16 ± 0.81	0.11
Color b* (yellowness)	11.45 ± 1.47	12.84 ± 0.94	12.88 ± 0.51	13.38 ± 1.32	12.31 ± 0.55	14.41 ± 0.79	0.45
Tenderness	227.41 ± 22.06	224.91 ± 24.61	230.92 ± 16.95	237.89 ± 12.05	261.26 ± 4.28	208.21 ± 17.13	0.44

Note: All values were expressed as mean ± SE; ^a,b,c^ values with superscript within row are significantly different at *p* < 0.05. T1: Negative control; T2: Positive control; T3: 25 mg/kg; T4: 50 mg/kg; T5: 75 mg/kg; T6: 100 mg/kg.

**Table 9:** Effect of *Y. schidigera* supplementation on the meat quality of broilers on day 42.

Parameters	Treatments						*p* Value
	T1	T2	T3	T4	T5	T6	
42 day-old (Finisher phase)							
Breast							
Cooking loss (%)	24.56 ± 0.43 ^a^	25.99 ± 1.06 ^a^	25.28 ± 0.67 ^a^	24.65 ± 1.14 ^a^	23.79 ± 1.15 ^a^	19.98 ± 0.14 ^b^	0.01
Muscle pH	5.67 ± 0.01 ^c^	5.68 ± 0.02 ^c^	5.73 ± 0.02 ^ab^	5.70 ± 0.01 ^bc^	5.72 ± 0.01 ^ab^	5.74 ± 0.01 ^a^	0.01
Drip loss at 24 h (%)	8.33 ± 0.31 ^ab^	8.07 ± 0.47 ^b^	9.63 ± 0.27 ^a^	8.71 ± 0.49 ^ab^	8.02 ± 0.05 ^b^	8.01 ± 0.01 ^b^	0.01
Drip loss at 48 h (%)	11.43 ± 0.11 ^a^	9.31 ± 0.27 ^bc^	10.08 ± 0.25 ^b^	9.75 ± 0.08 ^bc^	8.73 ± 0.24 ^c^	8.61 ± 0.37 ^c^	0.01
Color L* (lightness)	59.61 ± 1.01	58.98 ± 0.45	55.11 ± 1.88	60.01 ± 0.43	58.29 ± 1.46	59.85 ± 1.06	0.06
Color a* (redness)	2.21 ± 0.30	1.95 ± 0.23	2.47 ± 0.24	2.16 ± 0.37	1.61 ± 0.12	1.73 ± 0.42	0.36
Color b* (yellowness)	15.56 ± 0.34	16.21 ± 0.64	15.91 ± 0.14	15.93 ± 0.11	16.32 ± 0.57	15.97 ± 0.26	0.81
Tenderness	520.89 ± 14.11	520.20 ± 14.55	533.74 ± 10.15	526.85 ± 13.82	511.91 ± 8.78	520.08 ± 14.28	0.89
Drumstick							
Cooking loss (%)	26.72 ± 0.29 ^a^	25.46 ± 0.36 ^a^	26.75 ± 0.15 ^a^	24.10 ± 1.15 ^ab^	22.52 ± 0.51 ^b^	21.55 ± 0.88 ^b^	0.01
Muscle pH	6.19 ± 0.01 ^b^	6.20 ± 0.01 ^b^	6.20 ± 0.02 ^b^	6.20 ± 0.01 ^ab^	6.23 ± 0.01 ^ab^	6.25 ± 0.02 ^a^	0.01
Drip loss at 24 h (%)	7.17 ± 0.02	7.06 ± 0.11	7.08 ± 0.11	6.93 ± 0.13	6.75 ± 0.12	6.76 ± 0.13	0.05
Drip loss at 48 h (%)	7.51 ± 0.06 ^a^	7.38 ± 0.08 ^ab^	7.51 ± 0.06 ^a^	7.19 ± 0.02 ^bc^	7.04 ± 0.06 ^c^	6.98 ± 0.06 ^c^	0.01
Color L* (lightness)	58.12 ± 0.19	57.83 ± 0.12	56.89 ± 1.06	57.31 ± 1.25	59.42 ± 0.77	59.61 ± 0.71	0.13
Color a* (redness)	5.18 ± 0.41	3.76 ± 0.28	5.19 ± 0.37	4.92 ± 0.15	4.73 ± 0.69	3.71 ± 0.27	0.05
Color b* (yellowness)	13.14 ± 0.88	12.35 ± 0.65	11.13 ± 0.68	11.61 ± 1.38	9.85 ± 0.76	9.86 ± 0.79	0.09
Tenderness	279.54 ± 11.31	307.58 ± 15.84	273.42 ± 18.63	307.78 ± 20.73	284.08 ± 18.71	288.38 ± 14.84	0.61

Note: All values were expressed as mean ± SE; ^a,b,c^ values with superscript within row are significantly different at *p* < 0.05. T1: Negative control; T2: Positive control; T3: 25 mg/kg; T4: 50 mg/kg; T5: 75 mg/kg; T6: 100 mg/kg.
